# Supplementary material for: Salinity and nitrogen source affect productivity and nutritional value of edible halophytes
Source: PLoS One. 2023 Aug 15;18(8):e0288547. doi: 10.1371/journal.pone.0288547 (PMC10427017; doi:10.1371/journal.pone.0288547)
Supplement: S1 File — (PDF) [file pone.0288547.s001.pdf]

Supplemental Files

Supplementary Table 1: Macronutrients in the hydroponic solution at different ratios of NO_3^--N: NH_4^+-N

Nutrient	Inorganic Salt	NO_3^--N: NH_4^+-N					NO_3^--N: NH_4^+-N				
		1:0	0.75:0.25	0.50:0.50	0.25:0.75	0:1	1:0	0.75:0.25	0.50:0.50	0.25:0.75	0:1
		Salt concentration (mM)					Nutrient concentration (mg/L)				
N (Ca)	$Ca(NO_3)_2$	1.43	1.08	0.70	0.35	-	40 (57)	30 (43)	20 (28)	10 (14)	-
N	NH_4Cl	-	0.70	1.45	2.15	2.85	-	10	20	30	40
Ca	$CaCl_2.2H_2O$	-	0.35	0.72	1.07	1.43	-	14	29	43	57
K	K_2SO_4	0.50	0.50	0.50	0.50	0.50	40	40	40	40	40
Mg	$MgSO_4.7H_2O$	1.0	1.0	1.0	1.0	1.0	24	24	24	24	24
P	$NaH_2PO_4.2H_2O$	0.32	0.32	0.32	0.32	0.32	10	10	10	10	10
P	$KH_2PO_4.2H_2O$	0.32	0.32	0.32	0.32	0.32	10	10	10	10	10

Supplementary Table 2: ANOVA table of statistical analysis for the effect of hydroponic solution NaCl concentration and NO_3^--N: NH_4^+-N ratio (N source) on the shoot biomass, oxalate and nitrate concentration in ice plant and ruby saltbush.

Plant		Shoot biomass	Oxalate concentration	Nitrate concentration
Ice plant				
	Salinity	***	***	***
	N source	***	***	***
	Salinity x N	ns	*	***
Ruby saltbush				
	Salinity	***	***	***
	N source	***	***	***
	Salinity x N	**	***	***
