# Supplementary material for: Salinity and nitrogen source affect productivity and nutritional value of edible halophytes
Source: PLoS One. 2023 Aug 15;18(8):e0288547. doi: 10.1371/journal.pone.0288547 (PMC10427017; doi:10.1371/journal.pone.0288547)
Supplement: Attachment — Submitted filename: Response to Reviewers.docx [file pone.0288547.s002.docx]

Response to Reviewers.

The author(s) should perform appropriate statistical analyses (Tables 2 to 4) to improve the quality of the manuscript prior to sending to the reviewers for evaluating its suitability for publication.

The manuscript in its current format is unsuitable to review due to lack of statistical analysis. Data presented in the Tables 2 - 4 of the manuscript need to be further analyzed using appropriate statistics. Moreover, data presented in the Table 1 were not appropriate. Place the ANOVA information at the bottom of the table to avoid confusion. Once done, the manuscript needs to be resubmitted for review.

As suggested we have revised Table 1 and moved the ANOVA analysis results to Part B of the Figure.

We have also now included statistical analysis for Tables 2 to 4.

We have deleted Supplementary Table 2.
