# Supplementary material for: Salinity and nitrogen source affect productivity and nutritional value of edible halophytes
Source: PLoS One. 2023 Aug 15;18(8):e0288547. doi: 10.1371/journal.pone.0288547 (PMC10427017; doi:10.1371/journal.pone.0288547)
Supplement: Attachment — Submitted filename: review notes.docx [file pone.0288547.s003.docx]

The article titled “Salinity and nitrogen source affect productivity and nutritional value of edible halophytes” by Bronwyn et. al. is a unique piece of research and have merits of its own. However, as per observation of the reviewer, there are some queries about this article along with some decent scopes of modifications which need to be addressed as follows-

1. Please reframe the abstract by adding research methodologies and treatment combinations in brief. Only show key results in the abstract that are conclusive towards research objectives. Please also mention full forms of the abbreviations used, then use abbreviations in the text or abstract.
2. Please also mention in the abstract that there are two sets of experiments in this research, along with their treatment combinations and methodologies.
3. Please point out why there are two experiments in this research? Mention the research objectives clearly at the end of the introduction section (in relevance to the two sets of experiments). Please also mention which experiment fulfils which objective/s and how they interrelate with each other in terms of a conclusive remarks to this research.
4. Please mention the meteorological characteristics of the experimental site/s, along with the primary (before sowing) characteristics of the planting materials and irrigation water, especially in terms of soluble salt contents.
5. Please also mention growth phase duration and timeline for the plants under both of the experiments as well as growth phases and/or point of data collection for both plant and soil.
6. Please explain the rationale to harvest plat shoots at or near flowering stage (in line number 137).
7. Please mention the version of R-software.
8. Please arrange the entire result section objective wise.
9. Elaborate the Discussion section at least to satisfy the objectives. It is highly suggestive that the authors discuss each objective and at the end interrelate them to a conclusive remark.
10. Please explain tables and figures within the text. There are barely some mentions of those in the text.
