# Supplementary material for: Salinity and nitrogen source affect productivity and nutritional value of edible halophytes
Source: PLoS One. 2023 Aug 15;18(8):e0288547. doi: 10.1371/journal.pone.0288547 (PMC10427017; doi:10.1371/journal.pone.0288547)
Supplement: Attachment — Submitted filename: Review PONE-D-23-11250R1.docx [file pone.0288547.s004.docx]

Review for manuscript PONE-D-23-11250R1: Salinity and nitrogen source affect productivity and nutritional value of edible halophytes

Reviewer comments:

The manuscript is well written with minimal style and grammar issues (see below for minor cases). It covers subject material that would be of interest to readers of PLOS One. It covers halophytic plant responses to salt concentration as well as nitrate/ammonium additions. he manuscript makes a valid case that halophytic plants might be useful/productive as crops. The results/discussion/conclusions are reasonable and supported by the data and analysis. I would like to see some speculation from the authors on what kind for research is needed outside of a greenhouse setting.

Summary:

**GENERAL COMMENTS**

**Abstract:**

Well written and clear overall. Meets word limit guidelines. Abstract gives good overview of the project, the approach and conclusions.

P1 L21-22 – Provide taxonomic names (in italics) for ice plant and ruby saltbush.

P1 L23 Remove capitalization from ice plant and ruby saltbush.

**Introduction:**

P3 L56 Include taxonomic name for saltbush.

P3 L61 Comma after “poor bone growth”.

L97 to 99. Were oyster plant seeds verbalized prior to planting everything else or did vernalization start when the other seeds were planted? Make the sequence of events clear.

L99 – 103. Same comment as above with respect to ruby saltbush. Did those get planted in advance and then transplanted at the same time as other plants? Again, just make the sequence clear.

L115-126. Clarify planting practice sequence for each crop.

**Results**

The results and discussion seem reasonable to this reviewer.

L188 - 191 Figure caption is here (with no figure) and also with the actual figure shown after the references. It probably doesn’t need to be in the text since it is given with the figure.

**Discussion**

The discussion makes sense and flows well. No issues found.

**Conclusions**

The conclusions are supported by the data, results and discussion. One comment: this was a greenhouse experiment. Is there a need for studies in actual soils? For example, could these crops be options in soils where productivity (for more traditional crops) has declined due to salt accumulation from irrigation? It would be good to include some speculation on this in the discussion and/or conclusions.
