# Supplementary material for: Salinity and nitrogen source affect productivity and nutritional value of edible halophytes
Source: PLoS One. 2023 Aug 15;18(8):e0288547. doi: 10.1371/journal.pone.0288547 (PMC10427017; doi:10.1371/journal.pone.0288547)
Supplement: Attachment — Submitted filename: review notes revised BB June 27_TR_final.docx [file pone.0288547.s005.docx]

The article titled “Salinity and nitrogen source affect productivity and nutritional value of edible halophytes” by Bronwyn et. al. is a unique piece of research and have merits of its own. However, as per observation of the reviewer, there are some queries about this article along with some decent scopes of modifications which need to be addressed as follows-

1. Please reframe the abstract by adding research methodologies and treatment combinations in brief. Only show key results in the abstract that are conclusive towards research objectives. Please also mention full forms of the abbreviations used, then use abbreviations in the text or abstract.

The abstract has been rewritten as requested.

2. Please also mention in the abstract that there are two sets of experiments in this research, along with their treatment combinations and methodologies.

This has now been mentioned in the abstract.

3. Please point out why there are two experiments in this research? Mention the research objectives clearly at the end of the introduction section (in relevance to the two sets of experiments). Please also mention which experiment fulfils which objective/s and how they interrelate with each other in terms of a conclusive remarks to this research.

This has now been done. The following sentences have been added to the introduction to clarify the aims and relevance of each experiment: “*An initial glasshouse experiment investigated the growth (biomass) and nutrient uptake response of six edible halophytes to seven concentrations of NaCl in the irrigation solution. These results informed the salinity levels chosen for a second glasshouse experiment that investigated the effect of NO3- : NH4+ ratios in irrigation water on the growth and shoot oxalate concentration in two edible halophytes*.”.

4. Please mention the meteorological characteristics of the experimental site/s, along with the primary (before sowing) characteristics of the planting materials and irrigation water, especially in terms of soluble salt contents.

Relevant information was already mentioned in M and M.

5. Please also mention growth phase duration and timeline for the plants under both of the experiments as well as growth phases and/or point of data collection for both plant and soil.

This was revised in M and M.

6. Please explain the rationale to harvest plat shoots at or near flowering stage (in line number 137). This was done to ensure all material was at the same stage of maturity. We have now added this explanation in and around line 137
7. Please mention the version of R-software.

The R software version was 4.2.1. This has now been added to the first mention of this.

8. Please arrange the entire result section objective wise.

The results are arranged logically and follow the objectives of the study and the sequence of the two experiments.

9. Elaborate the Discussion section at least to satisfy the objectives. It is highly suggestive that the authors discuss each objective and at the end interrelate, them to a conclusive remark.
10. The other reviewer indicated the discussion flowed well and objectives and how they relate to the experiments have been discussed. We have tried to interrelate the key findings in the conclusion.
11. Please explain tables and figures within the text. There are barely some mentions of those in the text.

The information extracted from the tables specifically supports the objectives. Describing all the data in the tables would not be possible. We have extracted out the important information and referred to that.
